# Daily Deep Breathing Improves Psychological Symptoms and Cognition in Mild Alzheimer's Disease Patients

**DOI:** 10.1002/alz70858_098347

**Published:** 2025-12-25

**Authors:** Zihan Wang, Qiumin Qu, Ling Gao, Shan Wei, Xiaojuan Guo, Jin Wang

**Affiliations:** ^1^ The First Affiliated Hospital of Xi’an Jiaotong University, Xi'an, Shaanxi, China; ^2^ The First Affiliated Hospital of Xi'an Jiaotong University, Xi'an, Shaanxi, China; ^3^ The First Affiliated Hospital of Xi’an Jiaotong University, Xi’an, Shaanxi, China

## Abstract

**Background:**

It has been known that deep breathing could relieve emotions and accelerate blood circulation. Recent studies have shown that deep breathing could improve the glymphatic system through various mechanisms. However, the effects of deep breathing on Alzheimer's disease (AD) had not been determined.

**Method:**

We conducted **48 weeks blind assess clinical trial** involving persons 50 to 80 years of age with mild to moderate AD. Participants were randomly assigned in a 1:1 ratio to receive deep breathing (deep breathing with 15 cycles by 3 times a day) or conventional treatment. The primary end point was **the change from baseline at 48 weeks** in the score on the Alzheimer's Disease Assessment Scale‐Cognitive Subscale (ADAS‐cog), the Clinician Interview‐Based Impression of Change with Caregiver Input Scale (CIBIC‐plus), the Alzheimer's Disease Cooperative Study‐Activities of Daily Living Scale (ADCS‐ADL), and the Neuropsychiatric Inventory (NPI). Efficacy analysis was performed using **multivariate logistic**.

**Result:**

A total of 40 participants were enrolled, with 20 assigned to receive deep breathing and 20 to receive conventional treatment. Multifactorial logistic regression analysis revealed significant difference in the changes in **ADAS‐cog scores** (OR = 3.000, 95% CI: 1.090‐ 8.254, *p* = 0.033) and **NPI scores** (OR = 4.000, 95% CI: 1.337‐11.965, *p* = 0.013) between the deep breathing group and conventional treatment group. The changes in ADCS‐ADL scores (OR = 2.333, 95% CI: 0.897‐ 6.072, *p* = 0.082), MMSE scores (OR = 1.500, 95% CI: 0.613‐3.670, *p* = 0.374) and CIBIC‐plus scores (OR = 1.222, 95% CI: 0.506‐ 2.949, *p* = 0.655) had no significant difference between the two groups.

**Conclusion:**

During the 48‐week treatment period, patients with mild to moderate AD in the deep breathing group experienced **a slower decline in cognitive function** and had **milder psychiatric symptoms**. These results indicate that daily deep breathing exercises may offer a new approach for treating AD.

